# Imaging of hypoxia-inducible factor 1α and septin 9 interaction by bimolecular fluorescence complementation in live cancer cells

**DOI:** 10.18632/oncotarget.16527

**Published:** 2017-03-23

**Authors:** Maya Golan, Nicola J Mabjeesh

**Affiliations:** ^1^ Prostate Cancer Research Laboratory, Department of Urology, Tel Aviv Sourasky Medical Center, Sackler Faculty of Medicine, Tel Aviv University, Tel Aviv, Israel

**Keywords:** hypoxia-inducible factor 1α, septin 9, bimolecular fluorescence complementation, cancer, imaging

## Abstract

Hypoxia-inducible factor 1 (HIF-1) is a major mediator of the hypoxic response involved in tumor progression. We had earlier described the interaction between septin 9 isoform 1 (SEPT9_i1) protein and the oxygen-regulated subunit, HIF-1α. SEPT9_i1 is a member of the conserved family of GTP-binding cytoskeleton septins. SEPT9_i1 stabilizes HIF-1α and facilitates its cytoplasmic-nuclear translocation. We utilized split yellow fluorescent protein (YFP) bimolecular fluorescence complementation (BiFC) methodology to monitor the interaction between HIF-1α and SEPT9_i1 in live cells. N-terminal (YN) and C-terminal (YC) split YFP chimeras with HIF-1α and SEPT9_i1 on both their amino and carboxyl termini were generated. HIF-1α and SEPT9_i1 chimeras were expressed in cancer cells and screened for functional complementation. SEPT9_i1-YN and YC-HIF-1α formed a long-lived highly stable complex upon interaction. The BiFC signal was increased in the presence of hypoxia-mimicking agents. In contrast, YC-ΔHLH-HIF-1α chimera, which lacked the helix-loop-helix domain that is essential for the interaction with SEPT9_i1 as well as the expression of SEPT9_i1 252-379 amino acids fragment required for the interaction with HIF-1α, significantly reduced the BiFC signal. The signal was also reduced when cells were treated with 17-*N*-allylamino-17-demethoxygeldanamycin, an HSP90 inhibitor that inhibits HIF-1α. It was increased with fourchlorfenuron, a small molecule that increases the interaction between HIF-1α and SEPT9_i1. These results reconfirmed the interaction between HIF-1α and SEPT9_i1 that was imaged in live cells. This BiFC system represents a novel approach for studying the real-time interaction between these two proteins and will allow high-throughput drug screening to identity compounds that disrupt this interaction.

## INTRODUCTION

Hypoxia is a common finding in advanced human tumors and it is often associated with metastatic dissemination and poor prognosis [1]. The primary mechanism mediating adaptive responses to hypoxia is the regulation of transcription by hypoxia-inducible factor (HIF) 1 and 2 [2]. Cancer-specific HIF activity, especially in regions of intratumoral hypoxia, has been shown to mediate angiogenesis, epithelial-mesenchymal transition, stem-cell maintenance, invasion, metastasis, and resistance to radiation therapy and chemotherapy [3–5]. HIF-1 is a heterodimeric transcription factor composed of a constitutively expressed HIF-1β subunit and an oxygen-regulated HIF-1α subunit [6, 7]. Under normal oxygen conditions, HIF-1α is hydroxylated at specific proline residues (402 and 564), ubiquitinated by the tumor suppressor protein Von Hippel–Lindau (VHL) and targeted for proteasomal degradation. Under hypoxic conditions, hydroxylation is inhibited, and HIF-1α rapidly accumulates in the cytoplasm and translocates into the nucleus. HIF-1α then dimerizes with HIF-1β to recruit co-activators and drive transcription of many genes critical for key aspects of cancer pathogenesis [6, 8–10]. Increased levels of HIF-1α have been demonstrated in the majority of primary human cancers and their metastases [11–13], and they were shown to be related to poor prognosis and treatment failure [2]. Anticancer effects of HIF-1 inhibition have been evidenced in mouse models of human cancer (see review [2]). Considering the profound impact of HIF-1α on cancer progression, there has been vast growing interest in the biology of the HIF-1α pathway and in the development of direct or indirect HIF-1 inhibitors for cancer therapy [14–17].

Targeting specific protein-protein interactions that play central roles in the HIF system offer therapeutic possibilities [18]. We previously described an activation of HIF-1 pathway that was mediated by septin 9 isoform 1 protein (SEPT9_i1), a member of the mammalian septin family. SEPT9_i1 is a product of septin 9 variant 1 (SEPT9_v1) mRNA, originally designated as MSF-A [19]. This interaction increased HIF-1α protein stability and HIF-1 transcriptional activity *in vitro* and promoted proliferation, tumor growth and angiogenesis *in vivo* [19]. Knocking down SEPT9_i1 or disrupting HIF-1α/SEPT9_i1 interaction gave reciprocal effects: it led to the reduction of HIF-1 transcriptional activity and to decreased tumor growth and angiogenesis [20, 21]. Based on our accumulative data having indicated that this complex is important for tumor progression, we now aimed to target the HIF-1α and SEPT9_i1 interaction in the search for new inhibitors in the HIF-1 pathway. We chose to use a bimolecular fluorescence complementation (BiFC) assay that enables direct visualization of protein-protein interactions at high spatial resolution in live cells [22]. To design this BiFC assay, the yellow fluorescent protein (YFP) was split into two fragments (the N-terminal YN and the C-terminal YC) that are fused to the protein of interest (HIF-1α and SEPT9_i1). Reconstitution of the YFP fluorophore occurs when the two fragments of the split YFP are approximated to each other as a result of protein–protein interactions [23]. In this study, we established an *in vivo* binding assay for monitoring and imaging the intracellular localization of HIF-1α and SEPT9_i1 interactions in live cells. We showed specificity and validity of this assay using different genetic and pharmacological treatments to serve as a platform for screening new therapeutic compounds inhibiting HIF-1α/SEPT9_i1 interaction.

## RESULTS

### Generation of HIF-1α/SEPT9_i1 split-YFP system

**Figure 1:**
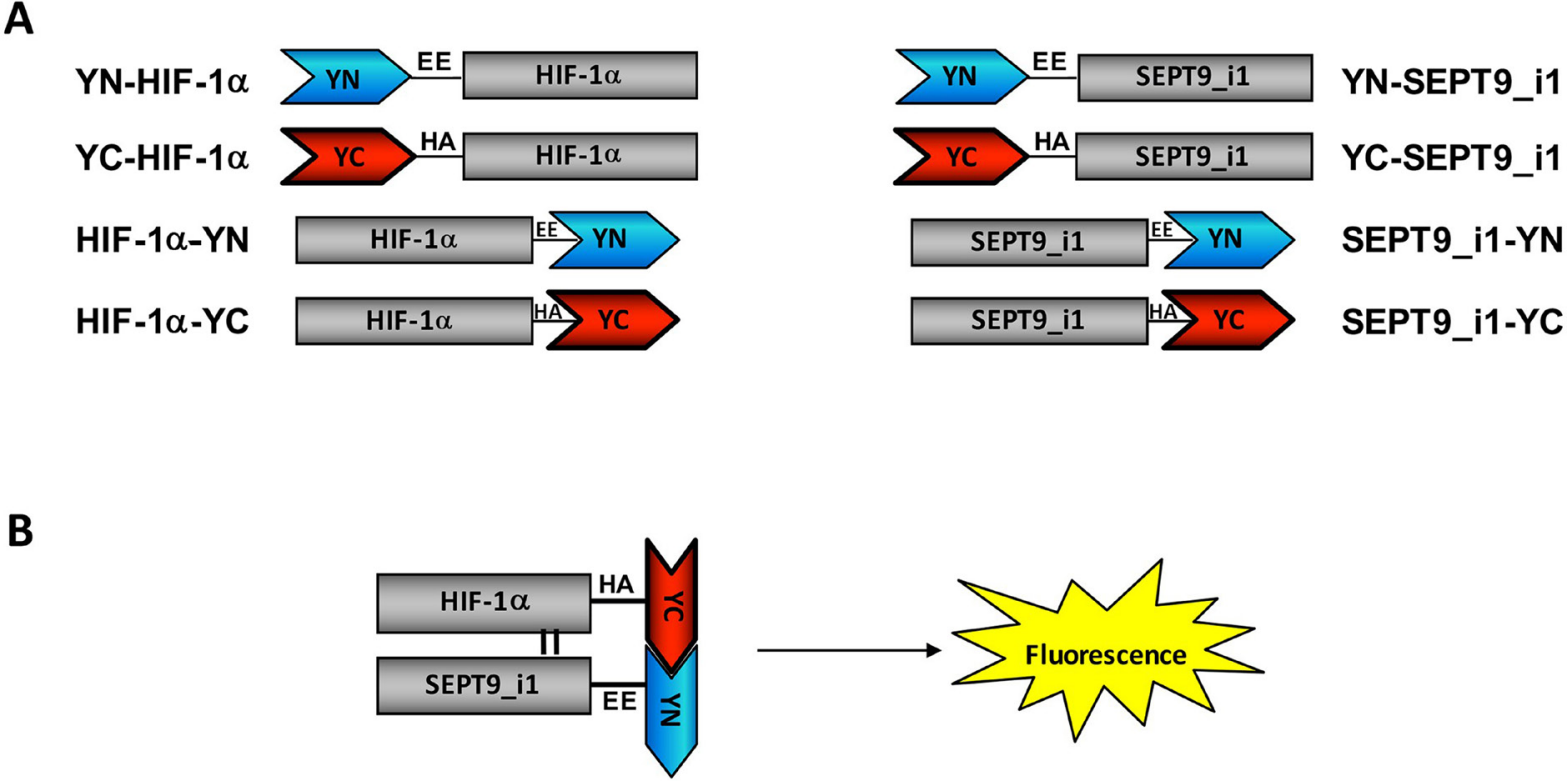
Construction of split-YFP HIF-1α and SEPT9_i1 chimeras (**A**) Illustration of HIF-1α and SEPT9_i1 split-YFP different chimeras containing a flexible linker (black line) with EE or HA tagging for YN and YC chimeras, respectively. The names of each chimera along with their schematic representation are shown. (**B**) A schematic presentation of the bimolecular fluorescence complementation (BiFC) principle: refolding and maturation of the complete YFP occur during the interaction of two complementary chimeras (YC-HIF-1α and SEPT9_i1-YN, in this case), and a fluorescence signal is accepted upon excitation.

**Figure 2:**
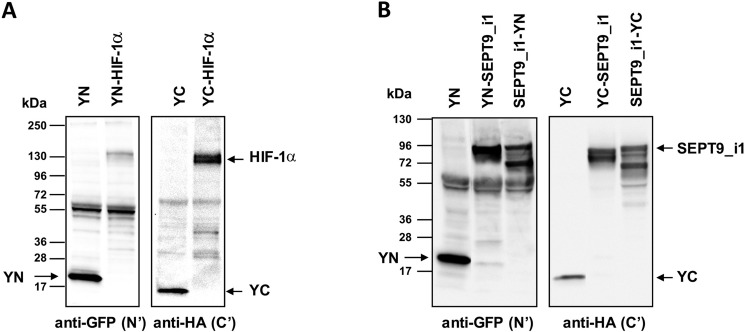
Expression of split-YFP HIF-1α and SEPT9_i1 chimeras HEK-293T cells were transiently transfected with the different split-YFP constructs. (**A**) Expression of split-YFP HIF-1α chimeras was analyzed by Western blotting, using anti-GFP-N’ for YN chimeras and anti-HA for YC chimeras, respectively. (**B**) The same as in (A) for SEPT9_i1split-YFP chimeras.

**Figure 3:**
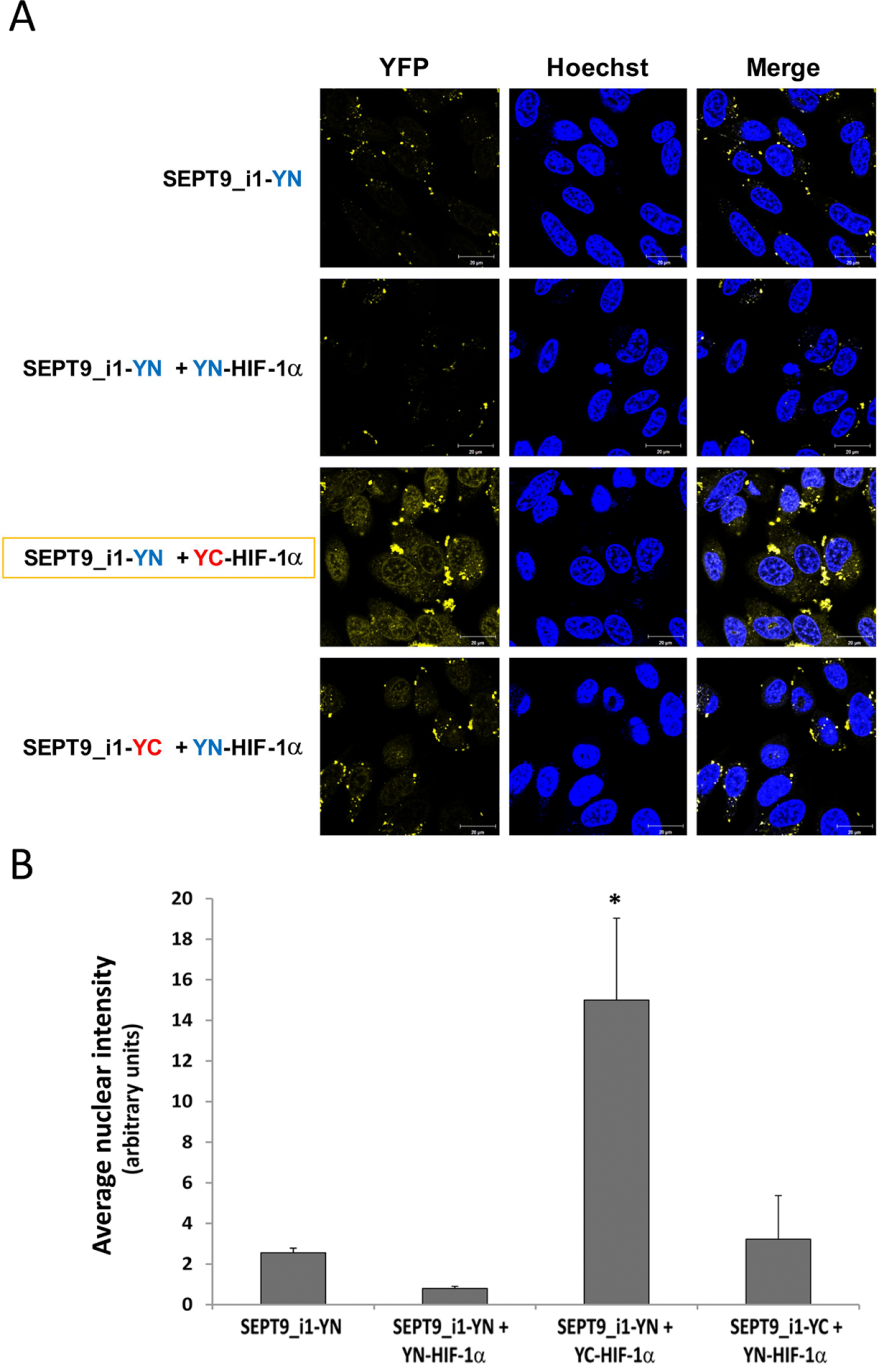
Optimal BiFC of split-YFP HIF-1α and SEPT9_i1 chimeras (**A**) PC-3 cells were seeded on glass-bottomed plates and transiently transfected with the indicated split-YFP constructs, using 10-fold more HIF-1α constructs (1 μg) than that for SEPT9_i1 constructs (0.1 μg), and treated overnight with 250 μM CoCl_2_ (mimicking hypoxia) prior to imaging. Forty-eight hours following transfection, the cells were imaged by confocal microscopy (magnification × 63; scale bars, 20 μm). Hoechst was employed for nuclear counter-staining (blue). The YC-HIF-1α/SEPT9_i1-YN pair produced the best signal (yellow). (**B**) Image analysis of the BiFC results shown in (A) comparing the nuclear staining intensity between the different pairs to the YN-SEPT9 control. The analysis was done on 3–8 different fields for each pair. *Bars*, SD; *P < 0.05 compared to each of the conditions.

We used YFP-based BiFC methodology in order to study the interaction between HIF-1α and SEPT9_i1 in live cancer cells. Split-YFP chimeras with either YN or YC on the N-terminal or C-terminal of each of the proteins were constructed as illustrated in Figure 1A. A yellow fluorescence signal is obtained after the interaction takes place (Figure 1B). The expression of HIF-1α‘s (Figure 2A) and SEPT9_i1′s (Figure 2B) chimeras was confirmed using Western blot analysis. Because HIF-1α's chimeras are susceptible to continuous degradation under normoxic conditions, more HIF-1α plasmids were used than SEPT9_i1 plasmids (10:1) when co-transfected. Before visualization, the transfected cells were treated with CoCl_2_, an iron chelator that mimics hypoxia and stabilizes HIF-1α under normoxic conditions [24]. All the different combinations of HIF-1α and SEPT9_i1 chimeras were tested for BiFC (data not shown). The pair combination that gave the best complementation signal wasYC-HIF-1α with SEPT9_i1-YN (Figure 3), and it was chosen for further studies. In some cases we noticed some speckles distributed mainly in the cytoplasm as in Figure 3. These speckles most likely appeared because overexpression of the chimeras that tend to aggregate and accumulate in p-bodies as proposed by Förg T. et al. [25]. To confirm that the selected chimeras are able to interact with each other YC-HIF-1α and SEPT9_i1-YN were expressed in PC-3 cells and processed for immunofluorescence labeling with antibodies to HA (YC-HIF-1α) (red), and GFP-N’ (SEPT9_i1-YN) (green) as well as with DAPI (blue) (Supplementary Figure 1). Image analysis showed 70% colocalization (Supplementary Figure 1). We also examined whether the two chimeras are transcriptionally active using a reporter gene assay expressing luciferase under hypoxia-response elements (Supplementary Figure 2). HIF-1 transcriptional activity was significantly induced by hypoxia and further increased in the presence of both chimeras (Supplementary Figure 2). These results indicated that the selected chimeras interact with each other as well as with HIF-1β to be transcriptionally functional.

### Characterization of YC-HIF-1α and SEPT9_i1-YN interaction in live cells

**Figure 4:**
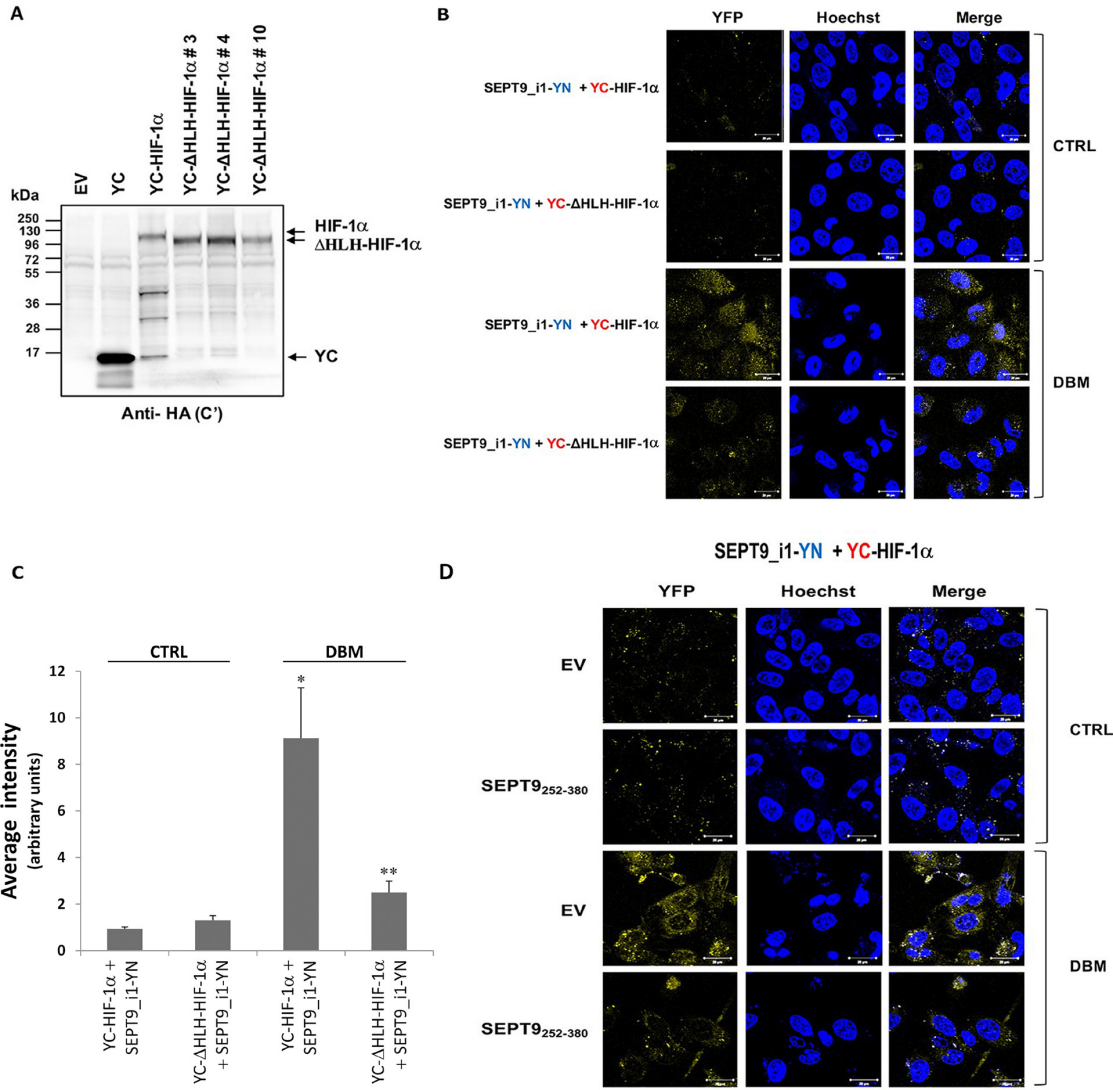
Disrupting YC-HIF-1α/SEPT9_i1-YN interaction decreases BiFC signal (**A**) HEK-293T cells were transiently transfected with pCDNA3.1(+) empty vector (EV), pCDNA3.1(+) with YC only (YC), YC-HIF-1α or YC-DHLH-HIF-1α constructs (clones #3, 4 and 10). After 48 hours, the cells were lysed and protein expression was analyzed by Western blotting using anti-HA antibody. (**B**) BiFC for PC-3 cells transiently transfected with YC-HIF-1α/YC-DHLH-HIF-1α and SEPT9_i1-YN or (**D**) with YC- HIF-1α and SEPT9_i1-YN together with SEPT9_i1 252-379aa fragment (the domain essential for the interaction with HIF-1α) or with its empty vector (EV). Cells were treated overnight with 100 μM DBM or with 0.01% DMSO as a vehicle control (CTRL) prior to imaging (magnification × 63; scale bars, 20 μm). (**C**) Image analysis of the BiFC results comparing the whole cellular staining intensity between the different pairs. The analysis was done on 3-9 different fields for each pair. *Columns*, average of the means, *Bars*, SD; *P < 0.01 compared with corresponding CTRL; **P < 0.05 compared to YC-HIF-1α and SEPT9_i1-YN with DBM.

We had previously identified the domains required for the interaction of HIF-1α with SEPT9_i1 as being the HLH domain of HIF-1α and amino acids 252–379 domain within the GTPase domain of SEPT9_i1 [26]. To confirm the specificity of the interacting chimeras, we designed and expressed YC-HIF-1α lacking the HLH domain (YC-DHLH-HIF-1α) with full-length SEPT9_i1-YN (Figure 4A). After transfection, the cells were incubated with another iron chelator dibenzoylmethane (DBM) [27], which induced a higher fluorescence signal than that of CoCl_2_ (not shown) upon complementation. The BiFC signal was significantly lower when YC-ΔHLH-HIF-1α was used compared with that of wild-type YC-HIF-1α (Figure 4B and 4C). To further characterize the specificity of the BiFC signal, we used co-expression of the domain of SEPT9_i1 (252-379aa) that is essential for the interaction with HIF-1α together with the active chimeras. SEPT9_i1 252-379aa fragment also reduced the BiFC signal obtained by YC-HIF-1α and SEPT9_i1-YN in the presence of DBM (Figure 4D). These results showed the specificity of the BiFC signal obtained from this system and re-confirmed our previous observations of the interacting sites of the two proteins.

### Pharmacological manipulation of YC-HIF-1α and SEPT9_i1-YN interaction

**Figure 5:**
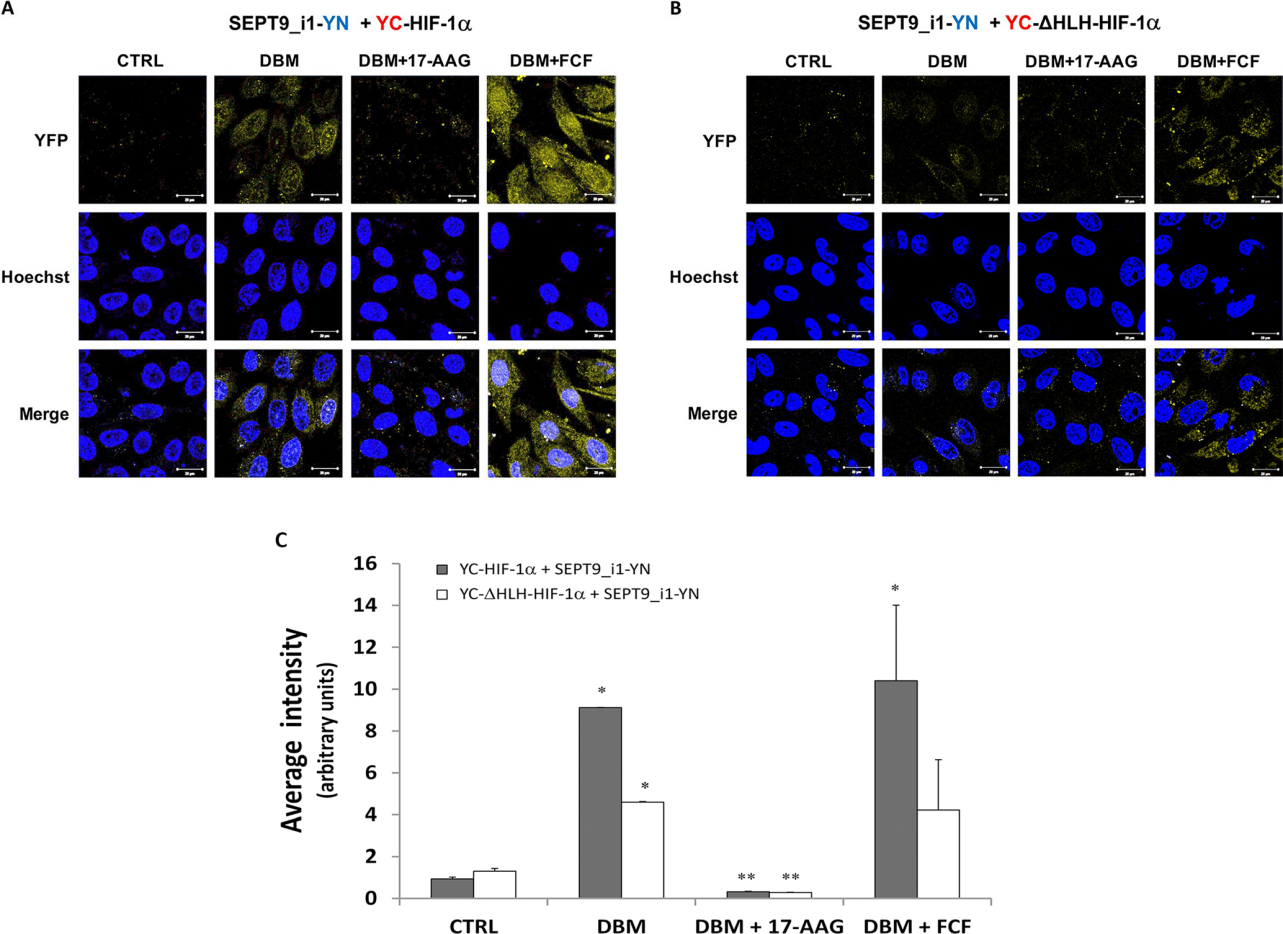
Pharmacological manipulation of YC-HIF-1α/SEPT9_i1-YN BiFC BiFC for PC-3 cells that were transiently transected with YC-HIF-1α (**A**) or YC-ΔHLH (**B**) together with SEPT9_i1-YN, and then treated overnight with 100 μM DBM alone, with 100 μM DBM and 1μM 17-AAG or 150 μM FCF, or with 0.01% DMSO as control (CRTL) prior to imaging (magnification × 63; scale bars, 20 μm). (**C**) Image analysis of the BiFC results comparing the whole cellular staining intensity between the different conditions. The analysis was done on 3-9 different fields for each pair. *Columns*, average of the means, *Bars*, SD; *P < 0.05 compared to corresponding CTRL; **P < 0.05 compared to corresponding DBM.

We then examined the ability of small molecule compounds to manipulate the interaction between HIF-1α and SEPT9_i1 in live cells. We used DBM that increases HIF-1α stability alone or together with the geldanamycin derivative, 17-N-allylamino-17-demethoxygeldanamycin (17-AAG), which is an HSP90 inhibitor known to decrease HIF-1α stability [28, 29], or with forchlorofenuron (FCF) that was previously shown to increase HIF-1α/SEPT9_i1 interaction *in vitro* [30] (Figure 5). As shown in Figure 5, 17-AAG decreased the BiFC signal, while FCF significantly increased the BiFC signal. All signals were much lower in the YC-ΔHLH-HIF-1α control transfected cells (Figure 5C). These results reconfirmed those of our previous study showing that FCF increased HIF-1α/SEPT9_i1 interaction and that this interaction can be manipulated using small molecule compounds.

### Optimizing YC-HIF-1α/SEPT9_i1-YN BiFC signal

**Figure 6:**
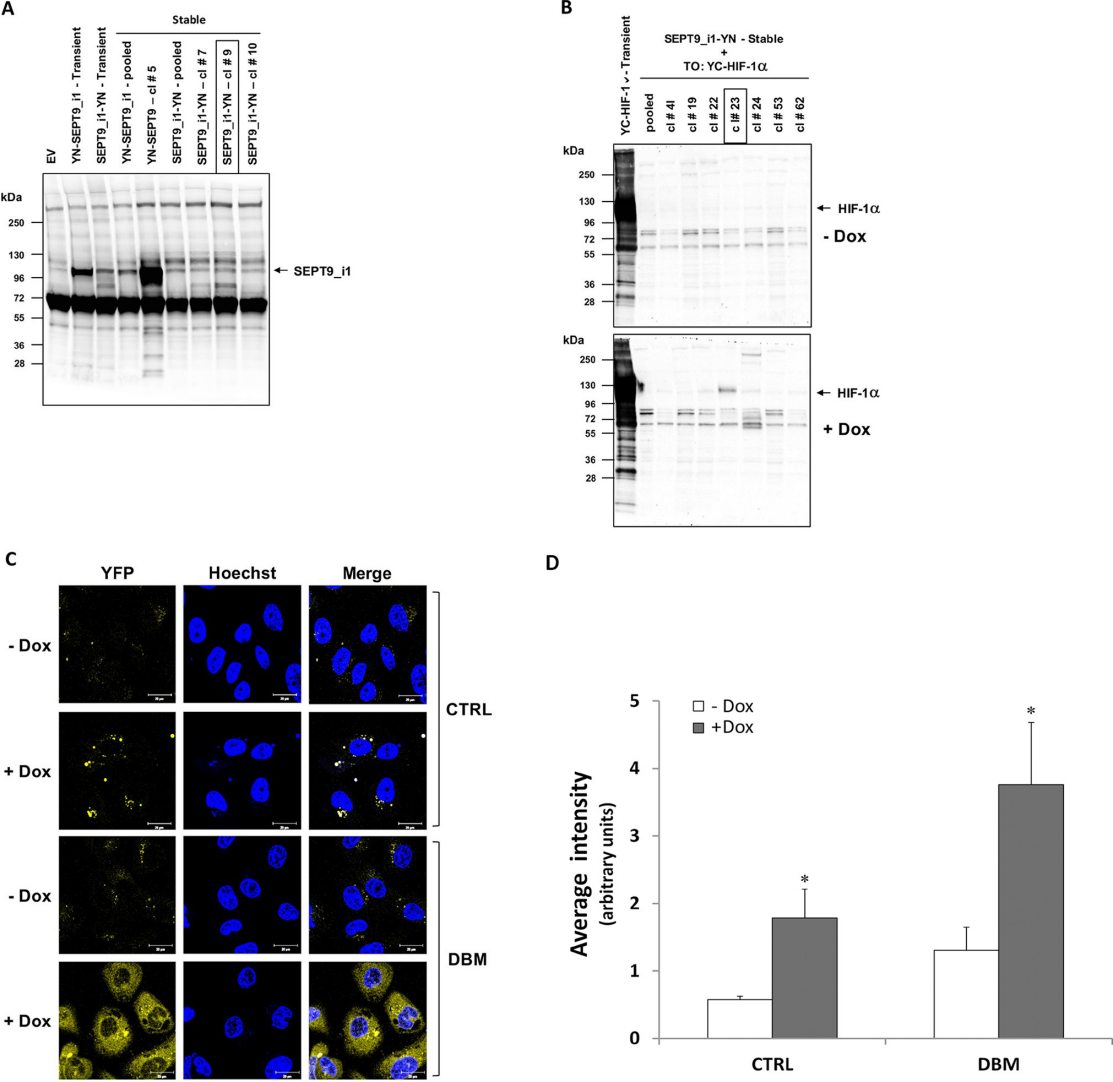
Stable expression of split-YFP SEPT9_i1-YN and inducible YC-HIF-1α increases the BiFC signal PC-3 cells were transfected with pCDNA3.1(+) vector expressing the YN-SEPT9_i1/SEPT9_i1-YN chimeras and selected with G418. (**A**) Expression of the YN-SEPT9_i1/SEPT9_i1-YN analyzed by Western blotting using anti-GFP-N’ antibody. SEPT9_i1-YN stable clone (clone #9) was chosen for further transfections. (**B**) SEPT9_i1-YN+Tet-On-YC-HIF-1α stable clones were untreated or treated with 1 μg/ml Dox for YC-HIF-1α induction and analyzed by Western blotting using anti-HA antibody. Transient transfection of the same constructs served as positive controls in both (A) and (B). (**C**) BiFC for PC-3 cells stably transfected with SEPT9_i1-YN and with Tet-On inducible YC-HIF-1α (clone #23), untreated or treated overnight with 1 μg/ml Dox for 3 days and with 100 μM DBM or 0.01% DMSO as control (CRTL) prior to imaging (magnification × 63; scale bars, 20 μm). (**D**) Image analysis of the BiFC results comparing the whole cellular staining intensity between the different conditions. The analysis was done on 3-9 different fields for each pair. *Columns*, average of the means, *Bars*, SD; *P < 0.05 compared to corresponding - DOX.

**Figure 7:**
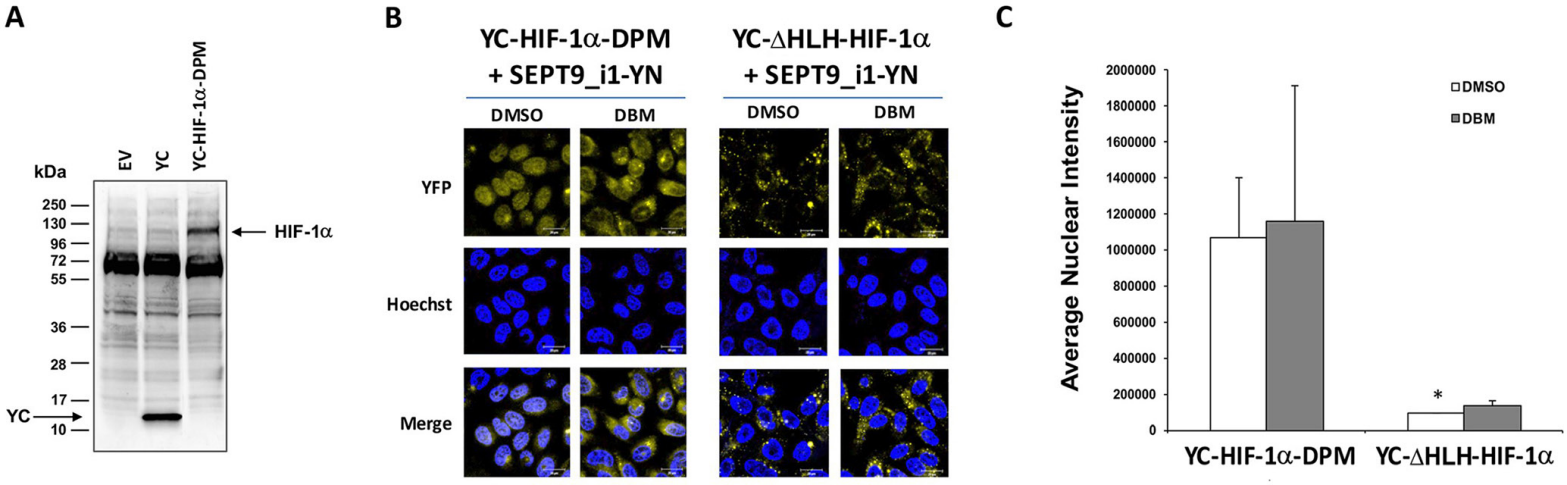
Double-proline mutated YC-HIF-1α increases nuclear BiFC signal (**A**) HEK-293T cells were transiently transfected with pCDNA3.1(+) empty vector (EV), pCDNA3.1(+) with YC only (YC) or with YC-HIF-1α-DPM constructs. After 48 hours, the cells were lysed and subjected to Western blot analysis using anti-HA antibody. (**B**) BiFC for PC-3 cells transiently transected with YC-HIF-1α-DPM or YC- HIF-1α-ΔHLH together with SEPT9_i1-YN were treated overnight with 100 μM DBM or with 0.01% DMSO as vehicle control prior to imaging (magnification × 63; scale bars, 20 μm). (**C**) Image analysis of the BiFC results comparing the nuclear and the whole cellular staining intensity between the YC-HIF-1α-DPM/SEPT9_i1-YN pair to the YC- HIF-1α-HIF-1α-ΔHLH/SEPT9_i1-YN pair, both with the DMSO vehicle control. *P < 0.05.

In order to increase the basal BiFC signal, we stably transfected PC-3 cells with SEPT9_i1-YN and Tet-On inducible YC-HIF-1α (Figure 6A), after which the cells were selected for YC-HIF-1α induction (Figure 6B). The BiFC signal of these stable cells was higher but only after DBM treatment (Figure 6C and 6D). Therefore, we next constructed HIF-1α mutated in its two proline hydroxylation sites (proline residues 402 and 564 within the oxygen-dependent degradation domain [ODDD]) termed double proline mutated (DPM) into the YC vector to create a YC-HIF-1α-DPM chimera (Figure 7A). This chimera was indeed stable under normoxic conditions independent of DBM and with a higher basal BiFC signal (Figure 7B). Moreover, image analysis showed that the BiFC signal was mainly nuclear and ~11-fold higher than the BiFC signal when YC-ΔHLH-HIF-1α was used (Figure 7C). We believe the use of the YC-HIF-1α/SEPT9_i1 BiFC system reflected real-time protein-protein interaction which can serve as a platform for further studies in the future.

## DISCUSSION

In the present study, we generated a novel BiFC system that imaged real-time interaction between HIF-1α and SEPT9_i1 proteins in live cancer cells (Figures 1, 2, 3). The system proved to be sensitive to pharmacological treatments that influenced the levels of HIF-1α protein or disrupted its interaction with SEPT9_i1 protein (Figure 5). This unique assay reconfirmed our previous finding of identifying the HLH part of HIF-1α and the GTPase part of SEPT9_i1 as the interacting domains required for the protein-protein interaction (Figure 4).

The overexpression of both HIF-1α and SEPT9_i1 has been correlated with tumor progression in numerous cancers. SEPT9 has been found to be overexpressed in diverse human tumors, including breast, head and neck, ovarian, endometrial, kidney, and pancreatic cancer [31–33], and its overexpression was recently shown to correlate with high-grade breast cancer [34] and prostate cancer [35]. HIF-1α up-regulation under normoxic conditions has been noted with increasing frequency in many cancers [36]. We had earlier shown that SEPT9_i1 upregulates HIF-1α in an oxygen-independent manner [21, 26]. Evidently, any interference of this interaction either by a specific shRNA to SEPT9_i1 [20] or by a SEPT9_i1 N-terminal peptide, which competes with SEPT9_i1 in the interaction with the nuclear transporter importin-α [21], decreases HIF-1 activation, tumor progression and angiogenesis.

Furthermore, when utilizing another mode of HIF-1α/SEPT9_i1 complex disruption such as reducing the dissociation of SEPT9_i1 filaments from the complex by FCF leads to the inhibition of the HIF-1 pathway and suppression of tumorigenic properties of prostate cancer cells [30, 37]. Therefore, we believe that disruption of the HIF-1α/SEPT9_i1 complex is beneficial for cancer therapy, and that the BiFC system is vitally important for evaluating novel compounds capable of disrupting the protein-protein interaction.

Using the double proline-mutated and nondegradable species of HIF-1α (Figure 7) adds an advantage to this system, especially in the search for molecules to disrupt the interaction per se rather than molecules that accelerate the degradation of HIF-1α. Secondly, this HIF-1α species is prevented from degradation under normal oxygen levels that are optimal for tumor cell growth. The BiFC system is highly sensitive and can be used in the future to portray the interaction in living animals. Compared with fluorescence resonance energy transfer (FRET) methodology, BiFC has a more stable signal and can be used *in vivo* [38].

Limitations of this system include anticipated difficulties to pass the cells after multistep transfections in large quantities until screening. In addition, fluorescent signals are very sensitive to minimal changes which may be caused by compounds with insignificant effects on HIF-1α/SEPT9_i1 interactions. It should also be taken into account that HIF-1α/SEPT9_i1 complex (BiFC signal) could be influenced by other cellular functions, such as the androgen-androgen receptor pathway, which does not exist in PC-3 prostate cancer cells. It is still encouraging to document changes after manipulating the interaction by using small molecule compounds, such as the HSP90 inhibitor 17-AAG, FCF, and DBM. This may reflect the reliability of the system.

In this study, we have established a novel BiFC system for studying and visualizing the interaction between the HIF-1α protein and the SEPT9_i1 protein. This assay represents a novel approach for studying and further investigating the real-time interaction between these two proteins in live cells. In addition, this system will allow high-throughput drug screening to identity compounds that disrupt the interaction between HIF-1α and SEPT9_i1. The distinct advantage of this system is that it will also allow target validation of identified compounds in the future.

## MATERIALS AND METHODS

### Cell culture

Human prostate cancer PC-3 cells were maintained in RPMI 1640 and human embryonic kidney cells (HEK 293T) were maintained in DMEM. All media were supplemented with 10% FCS and antibiotics. Cells were cultured at 37°C in a humidified atmosphere and 5% CO_2_ in air.

### Antibodies and reagents

The following primary antibodies were used: rabbit polyclonal antibody to SEPT9_i1, which was previously produced and characterized [19, 35], mouse monoclonal anti-HIF-1α (BD Biosciences, San Diego, CA), rabbit monoclonal anti-GFP (N-term) (Abcam, Cambridge, MA) and mouse monoclonal anti-hemagglutinin (HA) (Covance, Berkley, CA). Horseradish peroxidase conjugated secondary antibodies were used for Western blotting (Jackson ImmunoResearch, West Grove, PA). Dibenzoylmethane (DBM), 17-N-allylamino-17-demethoxygeldanamycin (17-AAG), forchlorfenuron (FCF) and doxycycline (Dox) were purchased from Sigma-Aldrich (St. Louis, MO).

### Plasmids construction

The human Flag-tagged HIF-1α, ΔHLH-HIF-1α (HIF-1α truncated at the HLH domain), HIF-1α-DPM (double proline mutation; HIF-1α mutated in prolines residues 402 and 564) and SEPT9_v1 used in this study have been described elsewhere [19, 26]. The split yellow fluorescent protein (YFP) constructs YN-EE, YC-HA, EE-YN and HA-YC [39] were a gift from S. Yalovsky and N. Ohad (Tel Aviv University, Israel) [40, 41]. YN-EE and EE-YN constructs contain a cDNA encoding for the N-terminal fragment of YFP (YN; residues 1–154 of YFP) fused to a 5-amino-acid linker (RSIAT), which, in turn, is fused to a 9-amino-acid EE tag. YC-HA and HA-YC constructs contain a cDNA encoding for the C-terminal fragment of YFP (YC; residues 155–238 of YFP) fused to a 17-amino-acid linker (RPACKIPNDLKQKVMNH), which, in turn, is fused to a 9-amino acid HA tag. YN-EE and YC-HA were cloned into pCDNA3.1(+) vector (Invitrogen, Carlsbad, CA), while EE-YN and HA-YC were cloned into pcDNA4A/Myc-His (Invitrogen) using PCR with the indicated primers and restriction enzymes (Table 1). Flag-tagged HIF-1α, ΔHLH-HIF-1α, HIF-1α-DPM and SEPT9_i1 [26] were re-cloned into these vectors and verified by sequencing (Table 1). Tet-On-induced HIF-1α was constructed by re-cloning YC-HIF-1α into a pcDNA5/TO vector (Invitrogen) (Table 1).

**Table 1:** Plasmids and primers used for constructs design

Construct name	Vector	Templates	Restriction sites	Tag	PCR primers
pcDNA3.1(+)-YN-H	pcDNA3.1(+)	*EcoRV, NotI*	YN-EE	EE	CGTAGATATCATGGTG AGCAAGGGCG
					CGTACCCGCGGCCGC TTCCATAGGCATATACT CTTCCTC
pcDNA3.1(+)-YC-H	pcDNA3.1(+)	*EcoRV, NotI*	YC-HA	HA	CGTAGATATCATGGCCG ACAAGCAGAAGAACG
					CGTACCCGCGGCCGCC GCATAGTCAGGAACAT CGTAAG
pcDNA3.1(+)-YN-S	pcDNA3.1(+)	*NheI, HindIII*	YN-EE	EE	CGTGGCTAGCATGGTGA GCAAGGGCG
					CGTAAAGCTT TTCCATA GGCATATACTCTTCCTC
pcDNA3.1(+)-YC-S	pcDNA3.1(+)	*NheI, HindIII*	YC-HA	HA	CGTGGCTAGCATGGCC GACAAGCAGAAGAACG
					CGTAAAGCTTCGCATAG TCAGGAACATCGTAAG
pcDNA4A-S-YN	pcDNA4A	*XbaI, PmeI*	EE-YN	EE	CGTATCTAGAGAAGAGG AAGAGTATATGCCTATG
					CGTAGTTTAAACTCACA TGATATAGACGTTGTGG CTGTTG
pcDNA4A-S-YC	pcDNA4A	*XbaI, PmeI*	HA-YC	HA	CGTATCTAGATACCCTT ACGATGTTCCTGAC
					CGTAGTTTAAACTTACTT GTACAGCTCGTCCATG
YN-HIF-1α	pcDNA3.1(+)-YN-H	*NotI, XbaI*	Flag-HIF-1α	EE	
YC-HIF-1α	pcDNA3.1(+)-YC-H	*NotI, XbaI*	Flag-HIF-1α	HA	
YN-SEPT9_i1	pcDNA3.1(+)-YN-S	*HindIII, XbaI*	Flag-SEPT9_i1	EE	
YC-SEPT9_i1	pcDNA3.1(+)-YC-S	*HindIII, XbaI*	Flag-SEPT9_i1	HA	
HIF-1α-YN	pcDNA4A-YN-S	*XbaI, PmeI*	Flag-HIF-1α	EE	
HIF-1α-YC	pcDNA4A-YC-S	*XbaI, PmeI*	Flag-HIF-1α	HA	
SEPT9_i1-YN	pcDNA4A-YN-S	*XbaI, PmeI*	Flag-SEPT9_i1	EE	
SEPT9_i1-YC	pcDNA4A-YC-S	*XbaI, PmeI*	Flag-SEPT9_i1	HA	
YC-ΔHLH-HIF-1α	pcDNA3.1(+)-YC-H	*NotI, XbaI*	Flag-ΔHLH- HIF-1α	HA	
TO-YC-HIF-1α	pcDNA5/TO	*EcoRV, XbaI*	YC-HIF-1α	HA	

HIF, hypoxia-inducible factor; SEPT9_i1, septin 9 isoform 1; HA, hemagglutinin; EE, E epitope; YN, N-terminal fragment of yellow fluorescent protein; YC, C-terminal fragment of yellow fluorescent protein.

### Transfection procedures

Cells were transiently transfected using Fugene transfection reagent (Promega, Madison, WI), according to the manufacturer's procedure. The amount of HIF-1α plasmids was 10-fold higher than that of the SEPT9_i1 plasmids used for transient co-transfections. PC-3 cells stably expressing split-YFP SEPT9_i1-YN and an inducible YC-HIF-1α were generated in three steps. First, the cells were transfected with SEPT9_i1-YN plasmid and the stable clones were selected and cultured in medium containing 100 μg/ml Zeocin (Bio Basic Canada, Canada), after which SEPT9_i1-YN expression in single clones was analyzed by Western blotting. Then, a selected clone stably expressing SEPT9_i1-YN was transfected with pcDNA6/TR (Invitrogen), and single clones were selected and cultured in medium containing both 20 μg/ml blasticidin S (In*vivo*gen) and 100 μg/ml Zeocin. Selected clones were transiently transfected with pcDNA5/TO-YC-HIF-1α. In order to select a single clone expressing the tet-repressor, the cells were untreated or treated with 1 μg/ml doxicycline for 72 hours and analyzed by Western blotting. Finally, selected clones expressing both SEPT9_i1-YN and tet-repressor were transfected with pcDNA5/TO-YC-HIF-1α. Stable clones were selected in media containing 250 μg/ml hygromycin (Invitrogen), 20 μg/mL blasticidin S and 100 μg/ml Zeocin. Inducible expression of YC- HIF-1α was confirmed by Western blotting.

### Protein extraction and Western blotting

HEK293 or PC-3 cells were analyzed as previously described [42]. Protein concentrations were determined using a bicinchoninic acid protein assay kit (Pierce, Rockford, IL), and 50–70 μg protein were subjected to SDS-PAGE followed by Western blotting. YN fragments were detected using anti-GFP-N’ antibody, and YC fragments were detected using anti-HA antibody, respectively. The signal was detected using an enhanced chemiluminescence kit (Clarity, Bio-Rad Laboratories, Richmond, CA) and digitally captured by MicroChemi (DNR Bio-imaging Systems, Jerusalem, Israel).

### Live cell fluorescence confocal microscopy

PC-3 cells were cultured on 35-mm glass-bottom plates and transiently transfected with the indicated plasmids. Forty-eight hours following transfection, the cells were imaged by a Zeiss LSM 710 confocal microscope for BiFC yellow fluorescence signal and for Hoechst nuclear staining. Laser and microscope settings were set to obtain optimal signals, following the manufacturer's instructions. Identical parameters (e.g., scanning line, laser light, contrast, and brightness) were used for comparing fluorescence intensities of the different conditions. From 4–6 microscopic fields were taken from each sample, and a representative field is shown in Figures. Western blotting analysis was performed to confirm the expression of each chimera or other proteins by. The expression of the chimeras and SEPT9_252–380_ was verified by immunoblottig using the respective antibodies; for SEPT9_252–380_ antibody to myc was used according to its c-terminal tag.

### Image and statistical analysis

Image analysis was performed using ImageJ software (http://rsb.info.nih.gov/ij). Hoechst staining was used to define the nuclear region of interest (ROI). Quantitative fluorescence data were exported from ImageJ-generated histograms into Microsoft Excel software for further analysis and presentation. Mean nuclear staining of all cells quantified from 4 different fields and under the different conditions was calculated and compared. The data are expressed as average of the means ± SD. When the number of speckles in the cytoplasm was high as in Figures 3 and 7, the quantitative analysis included only nuclear complementation signals based on DAPI staining. Student's *t* test was used to compare differences between the selected conditions. Statistical significance was set at *P* < 0.05.

## SUPPLEMENTARY MATERIALS FIGURES


