# Supplementary material for: Imaging of hypoxia-inducible factor 1α and septin 9 interaction by bimolecular fluorescence complementation in live cancer cells
Source: Oncotarget. 2017 Mar 23;8(19):31830–41. doi: 10.18632/oncotarget.16527 (PMC5458251; doi:10.18632/oncotarget.16527)
Supplement: Supplementary file 1 [file oncotarget-08-31830-s001.pdf]

Imaging of hypoxia-inducible factor 1α and septin 9 interaction by bimolecular fluorescence complementation in live cancer cells

Supplementary Materials

SUPPLEMENTARY METHODS

Immunofluorescence

Cells were plated on 12-mm glass coverslips (Fisher Scientific, Pittsburgh, PA) inserted into 6-well plates and allowed to attach overnight. Cells were fixed with cold methanol for 10 min and permeabilized by cold acetone for 3 min room temperature. Blocking was done in 1% BSA/10% normal donkey serum/PBS for 30 min at room temperature. Cells were subsequently incubated with primary antibodies to GFP-N' (1:100) and HA (1:100) diluted in primary antibody dilution buffer (Biomeda, Foster City, CA) for 2 h at room temperature. Secondary antibodies (Alexa Flour 594 donkey anti-mouse and 488 donkey anti-rabbit diluted 1:300 in PBS) were incubated for 1 h in the dark and DAPI was added for 10 min at room temperature. Samples were mounted onto slides using Fluorescent Mounting Medium (Golden Bridge International [GBI] Life Science Inc, Mukilteo, WA) and then examined under a Leica SP5 confocal microscope using a x63 NA1.4 lens. Laser and microscope settings were according to the manufacturer's instructions. Identical parameters (e.g., scanning line, laser light, contrast, and brightness) were used for comparing fluorescence intensities, and 3–9 microscopic fields were taken from each sample. Co-localization ratio was calculated using ImageJ software as follow: The area of each color staining (green or red) was selected and calculated separately. Then, the area in which the two colors co-localized was selected, using the ROI manger AND option, and calculated. The co-localization value was calculated as the percentage of the co-localization area (SEPT9_i1-YN + YC-HIF-1α) from the total YC-HIF-1α red staining area. The average value was calculated using 4 different fields.

Reporter gene assay

HIF-1 HRE-dependent luciferase activity was performed using the pBI-GL construct (pBI-GL V6L) containing six tandem copies of the VEGF hypoxia response elements. Cells were grown in 6-well plates and then transiently transfected in triplicate with DNA reporter plasmid together with YC-HIF-1α, SEPT9_i1-YN and corresponding empty vector , using GenePorter transfection reagent (Gene Therapy Systems, Inc., San Diego, CA), according to the manufacturer's instructions. Twenty-four hours after transfection, the cells were incubated either under normoxic or hypoxic conditions for 16 hours. The cells were lysed (0.1 M Kpi, pH 7.8, 0.2% Triton) and luciferase enzymatic activity was measured using a BMG Labtechnologies LUMIstar Galaxy Luminometer following the manufacturer's instructions.

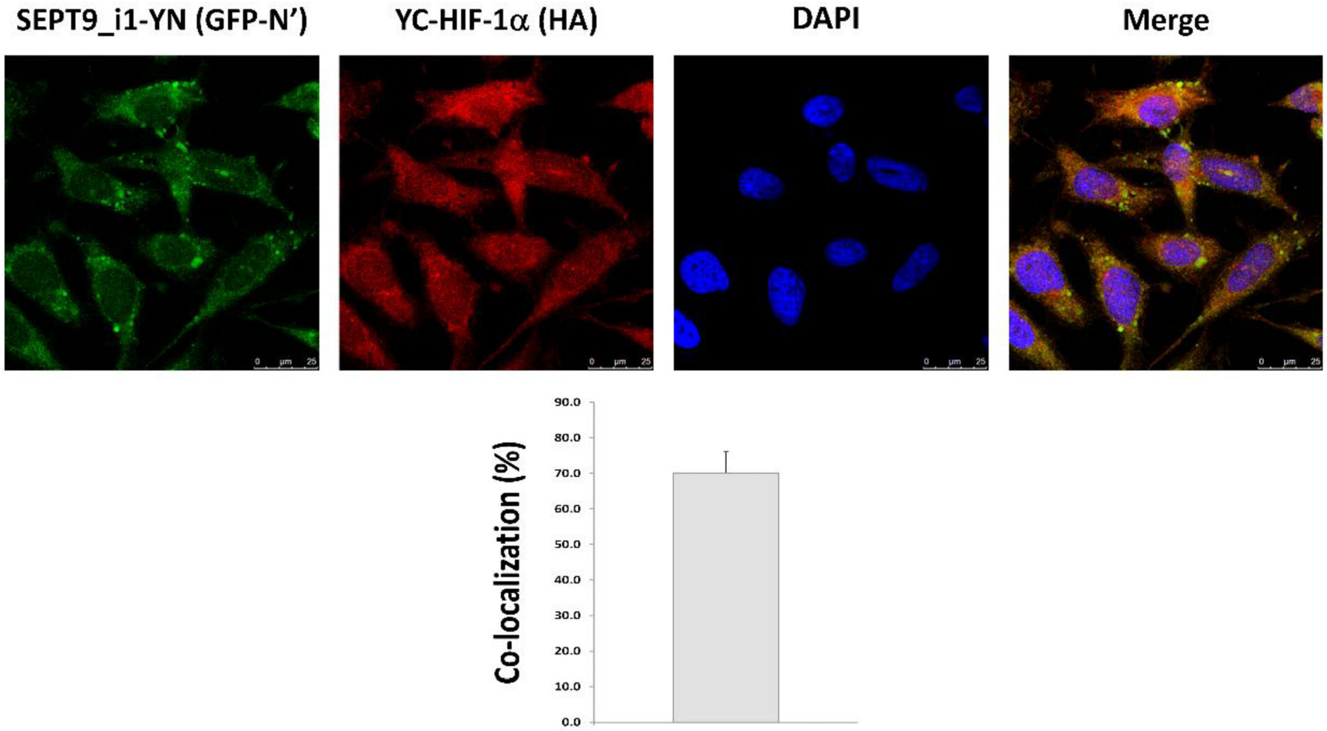

Supplementary Figure 1: YC-HIF-1α and SEPT9_i1-YN expression and co-localization by immunofluorescence labeling. PC-3 cells were transiently transfected with both YC-HIF-1α and SEPT9_i1-YN. Twenty-four hours following transfection the cells were grown under normoxic conditions. Forty-eight hours after transfection the cells were fixed and processed for immunofluorescence labeling with antibodies to HA (YC-HIF-1α) (red) and GFP-N' (SEPT9_i1-YN) (green) as well as with DAPI (blue). Staining was analyzed by confocal laser-scanning microscope (magnification × 63; scale bars, 25 µm). Representative cells from each treatment condition are presented. *Lower Panel*, Co-localization analysis for YC-HIF-1α and SEPT9_i1-YN staining using ImageJ software on 4 different fields of each sample. *Column*, average of the means; *Bar*, SD.

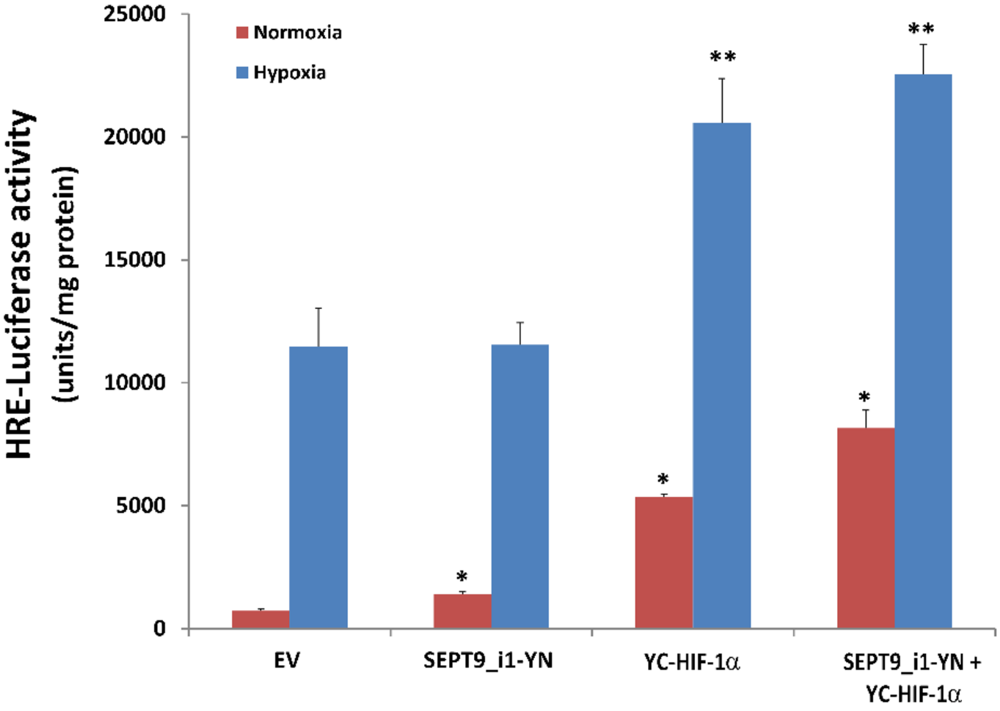

Supplementary Figure 2: YC-HIF-1α and SEPT9_i1-YN chimeras are transcriptionally active. PC-3 cells were cotransfected with 0.7 µg DNA of pBI-GL V6L expressing luciferase under the control of HRE and with 0.7 µg YC-HIF-1α, 0.7 µg SEPT9_i1-YN and 0.7 µg corresponding empty (EV) as indicated. After 24 hours of transfection, the cells were subjected overnight to normoxia or hypoxia and then analyzed for reporter gene assay. Arbitrary Luciferase activity units were normalized to the amount of protein in each assay point. *Columns*, mean ($n = 3$); *bars*, SD. *$P < 0.001$ compared with normoxic EV, **$P < 0.005$ compared with hypoxic EV.
